# Sex Differences Associated with Primary Biliary Cirrhosis

**DOI:** 10.1155/2012/610504

**Published:** 2012-05-30

**Authors:** Daniel S. Smyk, Eirini I. Rigopoulou, Albert Pares, Charalambos Billinis, Andrew K. Burroughs, Luigi Muratori, Pietro Invernizzi, Dimitrios P. Bogdanos

**Affiliations:** ^1^Institute of Liver Studies, King's College London School of Medicine, Denmark Hill Campus, London SE59PJ, UK; ^2^Department of Medicine, University of Thessaly Medical School, Viopolis, 41110 Larissa, Greece; ^3^Liver Unit, CIBEREHD, IDIBAPS, Hospital Clinic University of Barcelona, 08036 Barcelona, Spain; ^4^Department of Microbiology and Parasitology, Faculty of Veterinary Medicine, University of Thessaly, 43100 Karditsa, Greece; ^5^The Sheila Sherlock Liver Centre, and University Department of Surgery, Royal Free Hospital, London NW32QG, UK; ^6^Department of Clinical Medicine, Alma Mater Studiorum, Università di Bologna, Policlinico Sant'Orsola-Malpighi, 40138 Bologna, Italy; ^7^Center for Autoimmune Liver Diseases, Division of Internal Medicine, IRCCS Istituto Clinico Humanitas, 20089 Rozzano, Italy; ^8^Division of Rheumatology, Allergy and Clinical Immunology, University of California at Davis, Davis, CA 95616, USA; ^9^Department of Cellular Immunotherapy and Molecular Immunodiagnostics, Institute for Biomedical Research & Technology, 41222 Larissa, Greece

## Abstract

Primary biliary cirrhosis (PBC) is a cholestatic liver disease of autoimmune origin, characterised by the destruction of small intrahepatic bile ducts. The disease has an unpredictable clinical course but may progress to fibrosis and cirrhosis. The diagnostic hallmark of PBC is the presence of disease-specific antimitochondrial antibodies (AMA), which are pathognomonic for the development of PBC. The disease overwhelmingly affects females, with some cases of male PBC being reported. The reasons underlying the low incidence of males with PBC are largely unknown. Epidemiological studies estimate that approximately 7–11% of PBC patients are males. There does not appear to be any histological, serological, or biochemical differences between male and female PBC, although the symptomatology may differ, with males being at higher risk of life-threatening complications such as gastrointestinal bleeding and hepatoma. Studies on X chromosome and sex hormones are of interest when studying the low preponderance of PBC in males; however, these studies are far from conclusive. This paper will critically analyze the literature surrounding PBC in males.

## 1. Introduction

Primary biliary cirrhosis (PBC) is a chronic cholestatic liver disease characterised by an immunomediated inflammatory destruction of the small intrahepatic bile ducts, with fibrosis progressing to cirrhosis and subsequent liver failure [1–3]. The disease predominantly affects women (90% of patients) in middle age [1–4]. Studies from the United Kingdom suggest that PBC is the most frequent autoimmune liver disease, followed by autoimmune hepatitis and primary sclerosing cholangitis [5]. The incidence and prevalence of PBC appear to be rising in several countries [5–7]. Differences in the clinical course of the disease have been noted between Caucasian, African American, and Hispanic patients in the USA, with cirrhosis presenting more frequently in non-Caucasian patients [8]. Migration studies indicate that an individual's risk for PBC changes to be in accordance with the local population into which they move. This has led to the appreciation that environmental factors play an important role in the development of the disease [6, 9, 10].

Patients with PBC can be either asymptomatic with normal biochemistry tests or asymptomatic with abnormal biochemical blood tests, symptomatic, or finally can have advanced liver disease at the time of diagnosis [1–3]. Patients usually present in early stages, and the diagnosis of PBC is most often made when the patient is still asymptomatic with an abnormal cholestatic liver biochemistry and an immunological profile compatible with the disease which is discovered at a routine check [1–3]. Presenting symptoms frequently include fatigue, pruritus, and osteoporosis may be observed initially, in the absence of other signs of liver disease [1–3, 11–13]. The progression of PBC is usually slow paced, but symptoms of portal hypertension and hepatic decompensation (jaundice, ascites, or variceal bleeding) can develop several years after the initial diagnosis [1–3]. The current medical treatment of choice is with ursodeoxycholic acid, which appears to slow the disease progression and greatly improves the quality of life for many patients [14].

**Figure 1:**
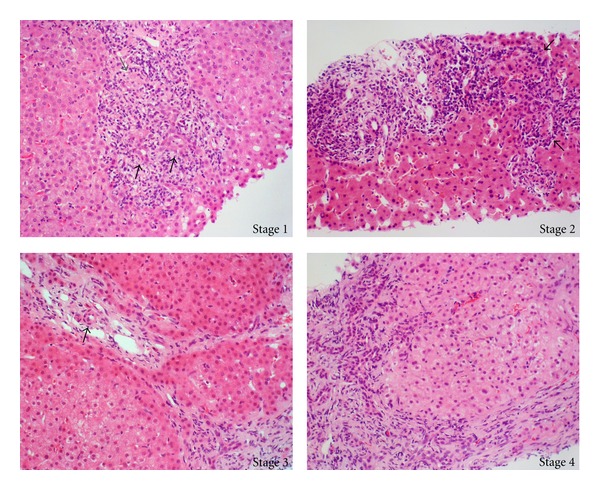
Histological staging in a representative case of a patient with primary biliary. Stage 1 reveals duct-centred inflammation showing chronic nonsuppurative destructive cholangitis (black arrows). A tiny granuloma is also seen (grey arrow). Stage 2 shows portal enlargement (arrows) with bile ductular reaction and inflammatory cell infiltration. Stage 3 is characterized by fibrous scaring bridging portal tracts with occasional foci of bile duct loss (no bile duct identified around an artery indicated by arrow). Stage 4 shows cirrhotic transformation.

The diagnosis of PBC is based on three widely accepted criteria: biochemical signs of cholestasis, seropositivity for disease-specific autoantibodies, and disease-characteristic histological features (Figure 1) [1–3]. PBC-characteristic histological features include destruction of biliary epithelial cells (BECs) and loss of small bile ducts with portal inflammatory cell infiltration and granuloma formation on occasion [1–3]. Cholestatic markers include increased levels of alkaline phosphatase (ALP) and gamma-glutamyl transferase (*γ*GT). IgM may be raised, and the most prominent immunological feature of the disease is the presence of high-titre antibodies against mitochondrial (AMA) and nuclear (ANA) antigens [1–3, 15–24]. AMAs do not appear to have clinical significance; however, disease-specific ANA can identify a subgroup of PBC patients with more advanced disease [15, 16, 24–36]. The presence of ANA at diagnosis seems to be able to identify individuals who will develop advanced disease faster than those seronegative for these autoantibodies [37]. It should be noted that patients may initially be found to be seronegative for AMA or disease-specific ANA with conventional techniques such as that of indirect immunofluorescence (IFL) [20, 38–40]. More sensitive tests using as antigenic source hybrids containing the major mitochondrial antigens have led to the appreciation that “true” AMA-seronegative cases may exist but are fewer than those considered in the past when conventional AMA testing was based on IFL and enzyme immunoassays based on the M2 antigen [20, 38–40].

PBC-specific AMAs are directed against components of the 2-oxo-acid dehydrogenase complexes, especially the E2 subunits of the pyruvate dehydrogenase complex (PDC-E2), branched-chain 2-oxoacid dehydrogenase complex (BCOADC), and 2-oxoglutarate dehydrogenase complex (OGDC) [1, 15, 16, 20, 36]. Anti-PDC-E2 AMAs are present in over 90% of AMA-positive cases [1, 15]. Isotypes of AMA may be IgG, IgA, and IgM, but high-titre AMAs of class IgG are found in up to 95% of patients [1, 15]. It has been demonstrated that the presence of AMA in the general population is much higher than the prevalence of PBC, indicating that AMA may precede the symptomatic onset of the disease [41], and studies have demonstrated that AMA-positive, asymptomatic patients often have histological features diagnostic of, or compatible with, PBC [41–44]. It is therefore considered that seropositivity for AMA is highly predictive of the development of PBC, which indicates the necessity for autoantibody screening and monitoring at regular intervals of close relatives of PBC patients and females in particular [36, 45]. However, the mechanisms responsible for the induction of PBC-specific AMAs and ANAs are not well defined [15, 46–51].

As mentioned, disease-specific ANA may have prognostic significance, which has raised the question as to whether diagnostic testing needs to incorporate assays for PBC-specific ANA [36]. Two ANA patterns may be observed on IFL: the “multiple nuclear dot” pattern mainly targets the nuclear body sp100 and promyelocytic leukaemia (PML) proteins and those giving the “nuclear membrane” (rim-like membranous) recognising gp210, nup62 and other nuclear membrane proteins [16–18, 36]. Both ANA types are present in 30% of PBC patients and can be present in AMA-positive and AMA-negative asymptomatic individuals and also in family members of PBC patients [16–18].

Genetic, epigenetic, environmental, and infectious factors have been considered important for the development of PBC and/or its progression from early stages to more advanced, life-threatening biliary epithelial cell destruction [33, 39, 47, 48, 53–73]. T cell dysregulation also appears to be a feature of PBC [74–79]. Molecular mimicry and immunological cross-reactivity involving homologous microbial and self-antigens have been considered as mechanisms responsible for the induction of disease-specific autoreactivity [33, 36, 50, 51, 58, 59, 80–93]. Genetic and environmental factors are likely involved, as indicated in twin and familial studies [53, 94–97]. Genome-wide association studies (GWASs) have allowed for the identification of genetic associations for PBC [39, 66, 67, 98–102]. *In vitro* studies have implicated antigen-specific B-, CD4, CD8 T-lymphocyte responses in the induction and/or maintenance of autoaggressive pathology [44, 47, 48, 61, 88, 89, 103].

Both the innate and the adaptive arms of the immune system have been considered important for the loss of immunological tolerance to AMA-specific PDC-E2 targets and the subsequent development of the disease [104–112]. As AMAs appear long before disease onset, investigators thought that antibody-dependent cellular cytotoxicity or antibody-antigen complement activation cell lysis mechanisms may lead to cholangiopathy. The ability of AMAs to inhibit the catalytic activity of PDC-E2 *in vitro* has further supported the pathophysiological role of AMAs [113]. PDC-E2 complexed with anti-PDC-E2 antibodies generate PDC-E2-specific cytotoxic cells, at a 100-fold lower concentration compared to the PDC-E2 alone [108, 109]. More recent studies have demonstrated the presence of immunologically intact PDC-E2 and other PBC-specific antigens in the surface of human intrahepatic biliary epithelial cell apoptotic bodies, making these antigens susceptible to antibody recognition, a feature which may also promote autoaggression [110–112]. The contribution of the innate and adaptive immune response in the development of PBC has been indicated in animal models resembling human PBC-specific immunopathology [47, 63, 65].

As mentioned, PBC is a disease which predominantly affects females, with the female-to-male ratios ranging from 9 : 1 to 22 : 1 [114, 115]. As such, few studies have taken into account PBC in men from both a pathogenic, as well as clinical viewpoint. The vast majority of PBC studies have been conducted on female patients. This review will examine the literature surrounding PBC in men, from a clinical and experimental standpoint (Table 1). We will also review the current literature which may, in part, explain why PBC rarely affects men and predominantly affects women.

### 1.1. PBC in Males: Case Reports and Epidemiological Studies

PBC occurring in males is relatively infrequent, which is reflected by the scarcity of studies examining PBC in males which are largely limited to case reports and small studies. The only twin study conducted in PBC consisted only of female pairs [95]. Brown and colleagues report PBC in a set of brothers, although the diagnosis in these early cases is questionable [116]. Tanaka et al. [117] notes two sets of brothers with PBC (one set in Britain and the other in France) in addition to several father-daughter and two brother-sister pairs. Unfortunately, the AMA status and clinical course of these patients are unknown [117]. Another set of brothers with a definitive diagnosis of PBC is followed up by the Barcelona group. Lazaridis and colleagues examined AMA status in 306 first-degree relatives of 350 PBC patients and found that AMA was present in 7.8% of brothers, 3.7% of fathers, and 0% of sons of PBC patients. It was not indicated whether the proband of the AMA-positive male's relatives were male or female [118]. The rarity of these case reports reflects the sporadic occurrence of PBC in males, which has also been demonstrated in epidemiological studies.

The female preponderance of PBC is reflected in several large epidemiological studies, and risk factors indicated in these studies tend to focus on those related to women [119–122]. A recent study by Corpechot and colleagues involved a French cohort of 222 PBC patients and 509 controls, all administered a questionnaire regarding demographic, lifestyle, and health factors [119]. In that study, 11% of the PBC patients were male, as were 15% of the control group. Several risk factors indicated include a history of recurrent urinary tract infection (rUTI), smoking, and family history of PBC, as well as oestrogen deficiency [119]. There were no risk factors indicated which were related to male sex [119]. A study by Prince et al. [122] involved two groups of PBC patients, one consisting of 318 patients from an epidemiological study, and the other group consisted of 2258 patients from a PBC support group [122]. The control group consisted of 2438 matched controls. Among PBC patients from the epidemiological study group, 8% were male, compared to 7% in the support group [122], which are similar rates to those found by another group [121]. Again, no specific risk factors were indentified for males, although it was noted in the study by Prince and colleague [122] that less than 1% of males had a history of hair dye use, compared to over 50% of females, which is of interest given that hair dyes have been implicated as a possible risk factor for PBC development. One of the largest epidemiological studies carried out included 1032 PBC patients from 23 tertiary care centres and 1041 controls, all administered a telephone questionnaire [120]. Among PBC patients, 7% were males compared to 8% of the control group. Again, there were no specific risk factors for males indicated [120]. These epidemiological studies have not identified any risk factors which are overtly male-specific, in contrast to several female-specific risk factors such as oestrogen deficiency. All studies demonstrated smoking and rUTI as risk factors [119–123], although it is unclear what proportion of males had a significant history of smoking or rUTI. As well, a recent study has indicated that rUTI occurs before PBC diagnosis in a large proportion of PBC patients but did not indicate what percentage of males had a history of rUTI [124]. The proportion of males with a history of rUTI warrants further investigation.

It is possible that mechanisms such as molecular mimicry with *E. coli* and other organisms are involved in both men and women [51, 123]. The development of experimental autoimmune cholangiopathy resembling PBC has been described in a 24-month-old C57BL/6 male mouse, after infection with *H. pylori* [125]. Liver histology demonstrated nonsuppurative, destructive cholangitis, peribiliary lymphoplasmacytic infiltration, and bridging fibrosis [125]. Serum antivacuolating toxin IgG levels were elevated in this mouse, compared to 13 other mice infected with *H. pylori* [125]. Those authors suggested that PBC may have developed due to molecular mimicry via anti-VacA antibodies [125]. However, Koutsoumpas and colleagues [126] highlighted that Goo et al. [125] did not specify if anti-PDC-E2 (AMAs) were present in the mouse. Further studies demonstrated insignificant homologous regions between *H. pylori* and PDC-E2 [126]. Inhibition studies abolished reactivity to PDC-E2 but not to VacA, and reciprocal studies using anti-VacA did not inhibit anti-PDC-E2 reactivity [126]. It was therefore unlikely that *H. pylori* infection was related to the development of PBC by mechanisms of molecular mimicry.

Exposure to environmental toxins has also not been studied in great detail, although this may be of particular interest in regards to PBC in men, based on occupational exposure. It has been noted that some clusters of PBC patients have higher rates of affected males compared to the general population, and some of these clusters are centred around coal mines and steel working industries, which are predominantly employed by men [68]. These observations may warrant further study as to the occupational exposures of men with PBC.

### 1.2. Clinical, Biochemical, and Histopathological Differences in Male versus Female PBC

 Clinical, biochemical, and histopathological differences in men versus women with PBC have been investigated in several early studies [52, 127, 128] (Table 1). One of these was conducted at the Armed Forces Institute of Pathology and involved 30 male and 30 age-matched female PBC patients, all AMA positive, with five of the 30 men being asymptomatic [127]. In addition to histological studies, symptoms and biochemical indices were also compared in each group. It was found that females experienced pruritus as a single symptom more often than males, in addition to experiencing more abdominal pain/discomfort as well as constitutional symptoms (malaise, anorexia, weight loss, fatigue, weight loss) [127]. In contrast, males experienced more jaundice, jaundice with pruritus, and upper gastrointestinal bleeding compared to females [127]. From a biochemical standpoint, ALP was slightly higher in symptomatic males compared to asymptomatic males, with both being higher than females in general [127]. Histologically, the only difference found was that symptomatic female patients had more piecemeal necrosis and that symptomatic males had more stainable copper storage than asymptomatic males [127]. Symptomatic females had more marked pseudoxanthomatous transformation than asymptomatic females [127]. That study concluded that there was little difference in male versus female PBC.

**Figure 2:**
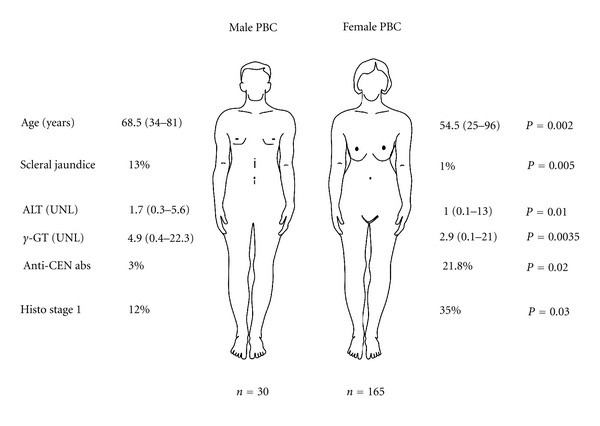
Clinical and Laboratory differences between women and men with PBC. The figure illustrates the significant differences between the Italian cohorts of men and women with PBC analysed by Muratori et al. [52] Only the parameters that reached statistically significant difference are given. More details are provided within the text; UNL, upper normal level; histo, histological; CEN, centromere; abs, antibodies.

 Another study, conducted by a specialist liver unit at King's College Hospital, compared the clinical and biochemical profiles of males and females with PBC, in addition to long-term outcome [128]. Thirty-nine (39) men and 191 women with PBC were enrolled, with the age of diagnosis and disease severity being similar in both groups. Pruritus was found to be less common in males than females (45% versus 68%), and it was suggested that female sex hormones may be linked with pruritus, as there was an increased frequency of women reporting pruritus beginning with the administration of oral contraceptives, as well as during pregnancy [128]. Much like the earlier study [127], the group at King's College Hospital found that gastrointestinal bleeding was more common among males patients (23%) than female patients (15%), although this was not indicated to be statistically significant [128]. In fact, the only statistically significant finding in regards to clinical signs was that females demonstrated skin pigmentation more often than males (55% versus 35%) [128]. Concomitant autoimmune diseases were also examined between the two groups. Sicca symptoms were present in 33% of females and 15% males, scleroderma in 13% of females and 8% of males, and Raynauds in 13% females and 3% of males [128]. These findings suggest that females were more likely to suffer concomitant autoimmune disease than males. Interestingly, that study was the first to report an increased frequency of type 2 diabetes in male PBC patients [128]. As well, men with PBC had a higher frequency of hepatoma, which has also been found in other studies [128–130]. No significant differences were observed in regards to AMA, biochemistry, histology, or survival [128]. An Italian study [52] compared clinical and serological data of 30 consecutive male and 165 female PBC patients (Figure 2). Histology was available in 83% of the males and 79% of females. Clinically, there was a significant difference in age between the two groups, with males presenting at a median age of 68.5 years compared to 54.5 years in females [52]. Scleral jaundice was more common among males (13%) than females (11%), which was the only statistically significant clinical difference among the two groups (Figure 2). However, it should be noted that 64% of males and 51.5% of females were asymptomatic at the time of diagnosis, although this was not statistically significant [52]. Biochemically, males had higher levels of ALT and *γ*GT [52]. Analysis of biopsy specimens revealed that stage I was present in 35% of females compared to 12% of males, although 36% and 28% of males were in stages III and IV, respectively, compared to 19% of females in both stages [52]. However, this was not found to be statistically significant. Immunological profiles regarding AMA and ANA were not different between the two groups, although a higher frequency of anti-centromere activity was noted in females (21.4%) than in males (3%) [52]. That study concluded that more advanced disease in males was most likely due to delayed diagnosis in this group, in which PBC is not initially suspected [52].

The studies above suggest that although there does not appear to be a significant difference in the biochemical, histological, and immunological profiles of males and females with PBC, symptomatology does appear to be different, with males being more likely to experience potentially life threatening complications such as upper gastrointestinal bleeding and hepatoma. It is not clear whether this is due to the advanced stage that these patients are presented due a delay in reaching a definite diagnosis of PBC.

### 1.3. Autoantibody Profiles in Men versus Women

 Although earlier studies addressed AMA positivity between males and females, none have examined the AMA specificities in men and women. Nalbandian and colleagues [114] conducted a study to determine if there were serological differences between men and women. That study involved 46 men and 42 women, all with high titer AMA [114]. Reactivity patterns were similar in both groups: PDC-E2 (72% males, 76% females), E3BP/Protein X (63% males, 62% females), BCOADC-E2 (41% males, 50% females), and OGDC-E2 (24% males, 17% females) [114]. Testing of the optical densities did not reveal any significant difference, which leads to the conclusion that there was no difference of AMA reactivity between males and females [114].

### 1.4. X Monosomy

 Given the female preponderance of PBC, the role of the X chromosome has been investigated [131, 132]. Links between the X chromosome and immunity have been strengthened by clinical data, such as IPEX and XSCID syndromes which are caused by mutations on Xp11 and Xq13.1, respectively [131]. As well, X chromosome defects are more frequent in women with late-onset autoimmune disease [60, 132–134]. Skewed X inactivation appears to be a feature of several autoimmune diseases which are concomitant with PBC, such as systemic sclerosis [135, 136], autoimmune thyroid disease [137], and Sjogren's syndrome [138], but not in PBC [139].

 Selmi also indicates that epigenetic factors, such as X chromosome inactivation, may also be involved in the development of PBC, as well as variable concordance rates of PBC among twins [95]. These theories are of interest, given the increased X chromosome monosomy rate in the peripheral lymphocytes of female patients with PBC [60, 133, 140]. Mitchell et al. [140] analysed 125 variable X chromosome inactivation status genes in peripheral blood mRNA and DNA from MZ discordant and concordant pairs. Consistently downregulated genes included CLIC2 and PIN4 in the twin with PBC, which was not found in the healthy twin or in control subjects [140]. Partial or variable methylation was found in both genes and did not predict transcript levels or X inactivation status [140]. That study demonstrates the complexity of epigenetic factors which must be considered not only in twin studies but also in the comparison of men and women with PBC.

### 1.5. Immunological Differences in Males and Females

 Immunological differences may also explain the variable rates of PBC between men and women [134]. For example, females demonstrate enhanced antibody production and cell-mediated responses following immunization [141], in addition to having an increased CD4 T-cell count [142]. Males show increased inflammatory responses to infectious organisms [143], and sex hormones appear to effect cytokine production, B-cell maturation, homing of lymphocytes, and antigen presentation, which is of interest given the association between hormone replacement therapy and PBC [134]. As well, sex hormones can affect immune cell functioning by binding to steroid receptors [144]. Oestrogen and androgen receptors are expressed on B cells, whereas CD8 T cells, monocytes, neutrophils, and NK cells express oestrogen but not androgen receptors [144]. One study has demonstrated that oestrogen treatment of peripheral blood mononuclear cells from SLE patients increased IgG production, as well as anti-dsDNA autoantibody levels [145, 146]. Testosterone was found to decrease IgG and IgM production by peripheral blood mononuclear cells in healthy males and females [147]. Despite these findings, it remains unclear as to what role sex hormones play in the pathogenesis of PBC, as well as the preponderance of PBC in females versus males.

## 2. Conclusion

 The reasons underlying the rarity of PBC in males remain elusive. Studies have failed to demonstrate significant differences in the biochemical, immunological and histological features of PBC in men and women. Clinically, there does appear to be some differences between the sexes, with females tending to present with pruritus more than males, and males are at higher risk of developing gastrointestinal bleeding and hepatoma. The reasons for this are unclear. Risk factors for PBC have been reasonably well defined, although these factors do not demonstrate any male-specific risk factors, such as occupational exposures, which is a point that needs to be addressed. As well, rUTI has been demonstrated as a risk factor in females, but the proportion of male PBC patients with a history of rUTI remains unknown. The role of sex hormones in the pathogenesis of PBC also requires further study, as this may also shed some light on why PBC is so rare in males.

## Figures and Tables

**Table 1:** Features of primary biliary cirrhosis (PBC) in men and women. Although PBC in men and women is largely similar, certain clinical features such as symptomatology and concomitant diseases differ between the sexes. Very little difference is noted in regards to histological or biochemical features, as well as antimitochondrial antibody (AMA) reactivity.

Feature	Comment
Age	(i) Men older than women

Histopathology	(i) Largely no difference observed (ii) More stage I in women than in men in one study (iii) One study notes more piecemeal necrosis and pseudoxanthomatous transformation in symptomatic females, and more stainable copper storage in symptomatic males

Symptomatology	(i) Abdominal pain, constitutional symptoms, and pruritus as a single symptom more common in females (ii) Jaundice as a single symptom more common in males

Biochemistry	(i) Slightly increased ALP in males *versus* females in one study; higher *γ*-GT and ALT in men than in women in another

Concomitant autoimmune or other diseases	(i) More females experienced Sicca symptoms, scleroderma, and Raynauds (ii) Increased incidence of hepatoma in males

AMA reactivity	(i) Similar antigenic reactivity patterns in males and females among studies

ANA reactivity	(i) Anticentromere antibodies more prevalent in women than in men in one study

## Abbreviations

ALP: Alkaline phosphatase
AMAs: Antimitochondrial antibodies
ANAs: Antinuclear antibodies
BEC: Biliary epithelial cell
*E.coli:*: *Escherichia coli*
*H. pylori*: *Helicobacter pylori*
PBC: Primary biliary cirrhosis
PDC: Pyruvate dehydrogenase complex
RA: Rheumatoid arthritis
SLE: Systemic lupus erythematosus
rUTI: Recurrent urinary tract infection.
